# An automated heart rate-based algorithm for sleep stage classification: Validation using conventional polysomnography and an innovative wearable electrocardiogram device

**DOI:** 10.3389/fnins.2022.974192

**Published:** 2022-10-06

**Authors:** Nicolò Pini, Ju Lynn Ong, Gizem Yilmaz, Nicholas I. Y. N. Chee, Zhao Siting, Animesh Awasthi, Siddharth Biju, Kishan Kishan, Amiya Patanaik, William P. Fifer, Maristella Lucchini

**Affiliations:** ^1^Department of Psychiatry, Columbia University Irving Medical Center, New York, NY, United States; ^2^Division of Developmental Neuroscience, New York State Psychiatric Institute, New York, NY, United States; ^3^Centre for Sleep and Cognition, Yong Loo Lin School of Medicine, National University of Singapore, Singapore, Singapore; ^4^Electronic and Information Engineering, Imperial College London, London, United Kingdom; ^5^Department of Biotechnology, Indian Institute of Technology, Kharagpur, India; ^6^Neurobit Inc., New York, NY, United States; ^7^Department of Pediatrics, Columbia University Irving Medical Center, New York, NY, United States

**Keywords:** artificial intelligence, bio-inspired algorithms, home testing, heart rate variability (HRV), sleep monitoring algorithm, sleep monitoring devices, sleep state classification, wearable devices and sensors

## Abstract

**Background:**

The rapid advancement in wearable solutions to monitor and score sleep staging has enabled monitoring outside of the conventional clinical settings. However, most of the devices and algorithms lack extensive and independent validation, a fundamental step to ensure robustness, stability, and replicability of the results beyond the training and testing phases. These systems are thought not to be feasible and reliable alternatives to the gold standard, polysomnography (PSG).

**Materials and methods:**

This validation study highlights the accuracy and precision of the proposed heart rate (HR)-based deep-learning algorithm for sleep staging. The illustrated solution can perform classification at 2-levels (Wake; Sleep), 3-levels (Wake; NREM; REM) or 4- levels (Wake; Light; Deep; REM) in 30-s epochs. The algorithm was validated using an open-source dataset of PSG recordings (Physionet CinC dataset, *n* = 994 participants, 994 recordings) and a proprietary dataset of ECG recordings (Z3Pulse, *n* = 52 participants, 112 recordings) collected with a chest-worn, wireless sensor and simultaneous PSG collection using SOMNOtouch.

**Results:**

We evaluated the performance of the models in both datasets in terms of Accuracy (A), Cohen’s kappa (K), Sensitivity (SE), Specificity (SP), Positive Predictive Value (PPV), and Negative Predicted Value (NPV). In the CinC dataset, the highest value of accuracy was achieved by the 2-levels model (0.8797), while the 3-levels model obtained the best value of K (0.6025). The 4-levels model obtained the lowest SE (0.3812) and the highest SP (0.9744) for the classification of Deep sleep segments. AHI and biological sex did not affect scoring, while a significant decrease of performance by age was reported across the models. In the Z3Pulse dataset, the highest value of accuracy was achieved by the 2-levels model (0.8812), whereas the 3-levels model obtained the best value of K (0.611). For classification of the sleep states, the lowest SE (0.6163) and the highest SP (0.9606) were obtained for the classification of Deep sleep segment.

**Conclusion:**

The results of the validation procedure demonstrated the feasibility of accurate HR-based sleep staging. The combination of the proposed sleep staging algorithm with an inexpensive HR device, provides a cost-effective and non-invasive solution deployable in the home environment and robust across age, sex, and AHI scores.

## Introduction

Sleep is a biological necessity that leads humans to spend roughly one third of their life asleep (Foster, 2020; Ramar et al., 2021). The circadian clock plays a crucial role toward ensuring that biological processes occur in the appropriate temporal sequence (Foster, 2020). Emerging research has highlighted the critical role that sleep plays in overall health (Grandner, 2017; Billings et al., 2020; Foster, 2020; Kocevska et al., 2021; Nochaiwong et al., 2021; Ramar et al., 2021) across the lifespan (Fatima et al., 2016; Bruce et al., 2017; Matricciani et al., 2019; Stores, 2022). Healthy sleep encompasses a variety of domains such as adequate sleep duration, appropriate timing, regularity, the absence of sleep disorders, and good quality (Grandner, 2017; Foster, 2020; Kocevska et al., 2021; Ramar et al., 2021). Poor sleep health has been associated with several negative health outcomes, including increased risk of cardiovascular disease (Gottlieb et al., 2006; Grandner et al., 2016), obesity (Fatima et al., 2016; St-Onge, 2017), depression (Alvaro et al., 2013; Murphy and Peterson, 2015; Nochaiwong et al., 2021; Nutt et al., 2022), and neurodegenerative disorders (Malhotra, 2018, 2022). Despite these well-known health risks, we are in the middle of a sleep crisis, with more than 70 million Americans experiencing sleep related problems (Colten and Altevogt, 2006) and with less than 20% of patients estimated to be properly diagnosed and treated for sleep disorders (Hossain and Shapiro, 2002). This sleep crisis had been further exacerbated by the COVID-19 pandemic. The terms “coronasomnia” or “COVID-somnia” (Gupta and Pandi-Perumal, 2020) have been introduced to describe the variety of symptoms of sleep dysfunction due to stresses related to fear of the virus itself or the psychosocial impact on daily living (Voulgaris et al., 2020; Becker, 2021; Nochaiwong et al., 2021; Bhat and Chokroverty, 2022; Stores, 2022).

Polysomnography (PSG) is presently considered the gold standard method to assess sleep and it is performed in sleep laboratories and clinical settings (Vaughn and Giallanza, 2008; Bianchi and Thomas, 2013; Bertoni and Isaiah, 2019; Stowe and Afolabi-Brown, 2020). It allows the characterization of sleep architecture and sleep disorders by the analysis of several physiological signals recorded simultaneously. Limitations of PSG include the cost of the devices, laborious setup procedures, the discomfort, and the necessity to have an expert perform the time-consuming process of coding the data (Hirshkowitz, 2016). While accelerometer-based actigraphy devices have been used in the field for decades as an alternative to PSG in patients’ living environment, estimation is limited to sleep/wake states thus, insufficient for a full evaluation of sleep architecture (Vulcan et al., 2021). These limitations highlight the need of noninvasive, inexpensive, and reliable sleep monitors that could be deployed in the home to monitor sleep health with automated solutions to perform reliable sleep stage scoring (Shah and Chircu, 2018; Bartoletti, 2019; Panch et al., 2019; Panesar, 2019; Adadi and Berrada, 2020).

Recent technological advancements have led to a proliferation of consumer-oriented tools to monitor sleep (Russo et al., 2015). Initially these tools relied mainly on activity, similarly to actigraphy, but lately they have started to incorporate additional physiological signals, such as EEG, heart rate (HR), breathing and pulse oximetry (de Zambotti et al., 2020; Fuller et al., 2020; Menghini et al., 2021). Increasing research indicates that HR and its variability might be an accurate and accessible physiological proxy for sleep measurement (Fonseca et al., 2017; Sridhar et al., 2020; de Zambotti and Baker, 2021; Li et al., 2021). A wealth of literature has shown profound differences in HR across sleep stages (Aldredge and Welch, 1973; Versace et al., 2003; Mendez et al., 2006), primarily comparing REM versus NREM sleep. In adults, REM sleep has been reported to be associated with an increment in low frequency power of the HR variability signal, and a decrement in high frequency power.

Currently, the vast majority of available nearables and wearables rely on contact photoplethysmography technology to estimate a continuous or pseudo continuous HR signal (Temko, 2017; Askarian et al., 2019; Jan et al., 2019; Kinnunen et al., 2020; Rocha et al., 2020, 2021). Technological advancement in non-contact sensor technologies are expected to bring a paradigm shift in healthcare monitoring. Most promising non-contact sensors utilize the signals acquired via ballistocardiography (Giovangrandi et al., 2011) or doppler radar sensors (Li et al., 2013) – both of which can be leveraged for extracting HR. Given these considerations, HR appears as a ubiquitous measurement across a variety of form factors, environments, and applications. Nonetheless, at present, ECG-derived HR is considered the gold standard in most applications. In this work, we validated the performance of an automated sleep staging algorithm that utilizes HR parameters derived from a single-channel ECG namely, Neurobit HRV (Neurobit Inc., New York, NY, USA). The algorithm solely relies on HR derived from the ECG signal thus, it results insensitive to the ECG morphology. As above stated, HR can be estimated at various degrees of accuracy. As such, the utilization of ECG-derived HR allow us to estimate the baseline performance of the sleep staging algorithm.

Additionally, the present work addresses another significant gap in the present state of the art. The results of automated sleep stage scoring derived from most of the currently available solutions lack validation against gold standard PSG measures. As such, the performance of these devices varies widely in independent validations (Chinoy et al., 2021). Moreover, access to the raw data is often limited and/or transparent access to the scoring algorithms is missing (Lee et al., 2019; Depner et al., 2020). Therefore, reproducibility and independent validation are severely restricted and unfeasible. This is further exacerbated by the fact that upgrades in firmware and scoring algorithms are not transparent to the users hence, limiting longer-term comparisons either within or between individuals or disabling the opportunity of leveraging historical open available dataset. To address these shortcomings, we provide transparent access to our cloud-based algorithms through a software development kit (SDK^1^) and an open application programming interface (API) along with reference code and documentation.^2^ The algorithms are versioned and openly accessible through the API.

In this manuscript, we performed validation on a public dataset [Physionet CinC (Goldberger et al., 2000)] and then on a secondary dataset with data acquired simultaneously with PSG and an ECG patch. Results demonstrate the feasibility of accurate sleep staging based on HR derived from ECG, obtained from either PSG recordings or wearable devices. The combination of the proposed sleep staging algorithm with inexpensive and commonly used ECG patches (∼100$) provides a cost-effective and non-invasive solution easily deployable in the home for large-scale sleep characterization in the field.

## Material and methods

### The sleep staging algorithm

**FIGURE 1:**
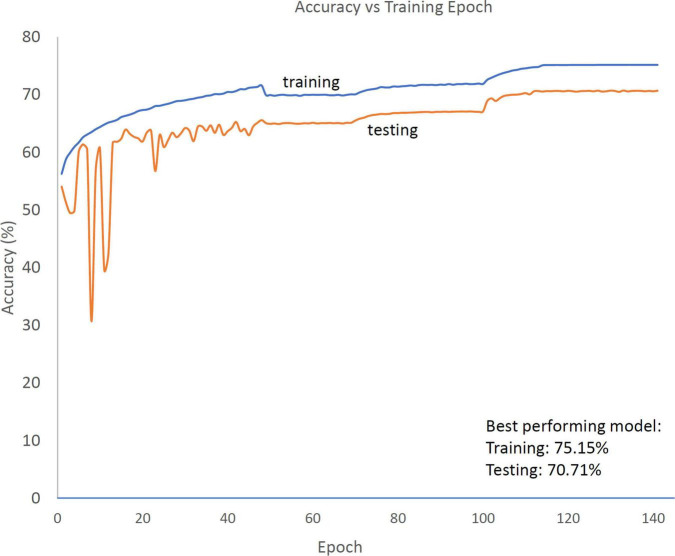
Trend of accuracy in the training (blue solid line) and testing (orange solid line) datasets as a function of training epochs for the 4-level model. Following an initialization period (for epoch value < ∼50), the difference in performance between the training and testing sets stabilizes at ∼5%.

The HR based automated sleep staging software called Neurobit-HRV was developed by (Neurobit Inc., New York, NY, USA). The deep-learning architecture was implemented in Python 3.6 using the Keras^3^ and Tensorflow 2 library.^4^ Firstly, the algorithm was trained and tested on private datasets comprising of ECG extracted from 12,404 PSG recordings collected at academic sleep centers in South-East Asia (35%), North America (30%) and Europe (30%). Fifty-nine percent of the total assessed participants had a suspected sleep disorder whereas the remaining 41% was contributed by healthy subjects. The mean age of such aggregated dataset was 42.3 ± 16.8 (mean ± std) years. The observations were randomly split into training (80%) and testing (20%) data at the participant level, stratified by the data source. The model which obtained the smallest test error was selected as the optimal one. Once the optimal model was obtained, the model weights were frozen and made available via a versioned API. This API is publicly available, and it was employed for the purpose of validating the performance of the algorithm as described in the following. Figure 1 displays the trends of model accuracy for the training and testing sets as a function of progressive iterations. The software can operate on either a single channel ECG or directly on R-peak locations, given it leverages the HR signal. To achieve optimal performance, the temporal precision of the R-peak location must be ± 4 ms or better. Neurobit-HRV incorporates an extensive wavelet-based ECG signal quality assessment toolbox for a real-time QRS detector, followed by a spurious R-peak detector for signal processing and quality assurance. Then, the processed RR interval tachogram is fed to the above-described automated sleep staging algorithm. Sleep state classification can be performed with different levels of granularity, namely 2-level (Wake; Sleep), 3-level (Wake: NREM: N1+N2+N3; and REM) or 4-level (Wake; Light: N1+N2; Deep: N3; and REM) in 30 s epochs compliant with the American Academy of Sleep Medicine (AASM) standard. The 2- and 3- level classifications are obtained by appropriately collapsing the 4- level classification. As such, the figures of merit for the classification of segments labeled as Wake are identical across models, while in the 3-level model, Light and Deep Sleep segments from the 4-level model are collapsed and classified as NREM segments. Additional information describing the mode of operation of the algorithm are reported in the Supplement (see Supplementary Figure 1).

### Participants and study protocol

#### You Snooze You Win – The PhysioNet Computing in Cardiology (CinC) challenge 2018 dataset

The CinC dataset is available at: https://physionet.org/content/challenge-2018/1.0.0/ and it is comprised of 994 participants (18–90 years old) which were monitored at Massachusetts General Hospital (MGH) sleep laboratory for the diagnosis of sleep disorders. Each participant has a complete set of 30-s annotated segments with corresponding sleep stages and respiratory events annotated by clinical staff at the MGH according to the AASM manual for the scoring of sleep. For this study, the initial preprocessing step consisted of assessing the quality of the ECG recordings extracted from the ensemble of recorded signals (EEG, EOG, EMG, respiration, and SaO_2_). The signal quality index is an estimate of signal to noise ratio (SNR). The ECG channel was fed to the Neurobit-HRV software to extract the RR interval tachogram along with a signal quality index. A proprietary wavelet-based technique was utilized to isolate the ECG signal from the background noise. The amplitude (A) of the ECG signal was computed via maximum filtering over windows of 3 s. The amplitude of the background noise was calculated as √2*root mean square (RMS) amplitude of the background signal over windows of duration 10 s. The signal to noise ratio is calculated according to Equation 1:


$$\text{SNR}=20\,\log\left(\frac{A_{\text{signal}}}{A_{\text{noise}}}\right)\,\text{dB}$$

By progressively sliding the 3-s and the 10-s windows (one sample at the time), a continuous SNR signal was computed for each epoch to be scored. An epoch is defined as rejected if 50% or more samples have an SNR value < 5 dB. A recording was excluded from the processing steps described in the following if 10% or more of the epochs were rejected. Illustrative examples of rejected epochs are included in the Supplementary materials (see Supplementary Figure 2). For the retained recordings, all epochs – including those with an associated SNR below the illustrated rejection criteria – were scored.

#### Z3Pulse datasets

The Z3Pulse dataset consisted of a set of 52 healthy adults in the age range of 23–69 years with no known sleep or psychiatric disorders. All participants provided written informed consent in compliance with a protocol approved by the National University of Singapore’s Institutional Review Board (NUS-IRB) and were compensated for their participation. The study protocol was carried out over three nights, when trained research assistants visited participants’ homes. On each night, participants wore a wearable ECG patch paired with an Android phone, alongside with simultaneous PSG. PSG was collected using the SOMNOtouch device (SOMNOmedics GmbH, Randersacker, Germany). Electroencephalography was recorded from two channels (C3 and C4 in the international 10–20 system of electrode placement) referenced to the contralateral mastoids. The common ground and reference electrode were placed at Fpz and Cz, respectively. Electrooculography (EOG; right and left outer canthi) and submental electromyography (EMG) were also recorded. EEG signals were sampled at 256 Hz and impedance was kept at less than 5KΩ for EEG and below 10KΩ for EOG and EMG channels. Sleep scoring was manually performed based on the AASM manual (Silber et al., 2007). Self-reported bed and wake up times were also collected. Each participant had at least one usable recording, 40 (∼77%) had 2 separate overnight recordings and 19 (∼37%) had 3.

**FIGURE 2:**
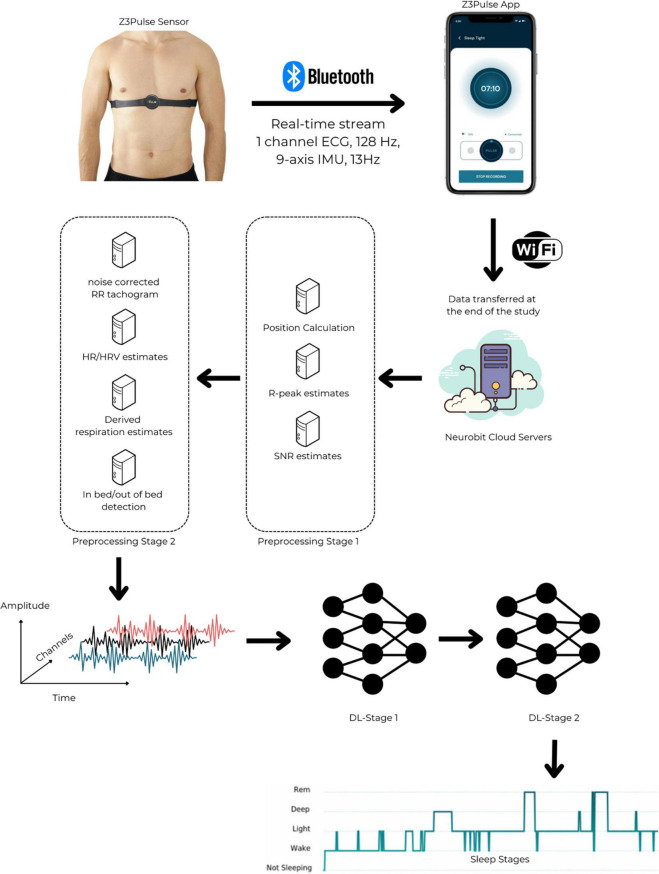
*The Z3Pulse system and processing pipeline – from data acquisition to automated sleep stage scoring.* The Z3Pulse sensor is a chest-worn wearable. It is secured by a one-time Ag-Cl patch or by a reusable belt. It transmits a single channel ECG and inertial measurement unit data in real-time to the Z3Pulse App. The app is operated via a user-friendly interface and stores the data locally for the entire duration of a recording. Once a given overnight sleep study is terminated, the collected data is transmitted to the Neurobit Cloud Servers. The data undergoes two stages of pre-processing. In the Preprocessing Stage 1, the analytics mounted on the Neurobit Cloud Servers enable the calculation of sleep position and detection of R-peaks (alongside the associated SNR values). Subsequently, these derivatives are pushed forward into the pipeline (to the Preprocessing Stage 2). At this stage, the noise-corrected RR tachogram, HR, HRV, the derived respiration estimates are calculated. Additionally, leveraging the position data (derived at the Preprocessing Stage 1), the in bed/out of bed score if obtained for each available/accepted epoch. The time-series data originated at the Preprocessing Stage 2 is then fed to two-stage deep-learning models. In a nutshell, the deep-learning model is based on a temporal convolutional model (Lea et al., 2017) with inception-residual networks (Szegedy et al., 2017). The first stage (DL-Stage 1) has 492,420 parameters and the second stage (DL-Stage 2 has 538,796 parameters. These networks use temporal convolutional networks along with inception layers. Overall, the two stages combined have a total of 1,031,216 parameters. Benefiting from the users’ feedback, an updated version of the pipeline mounts the Preprocessing Stage 1 is directly onto the device. Such processed data is stored locally in device memory. These results are transmitted via Bluetooth to the Z3Pulse App when a given recording is terminated. This updated architecture virtually prevent any data loss due to disconnections of the Z3Pulse Sensor from the Z3Pulse App during an overnight sleep recording.

The wearable ECG patch utilized in this study was the Z3Pulse device (Neurobit Inc., New York, USA), a chest worn, wireless device capable of recording HR, body position, activity and temperature. Z3Pulse was developed using the Movesense open wearable tech platform (Movesense, Finland^5^). The device is first connected to a reusable belt worn around the chest or a one-time Ag-Cl patch. In the latter case, the patch is directly applied on the skin below the sternum. The Movesense device consists of a single-channel ECG sensor, a nine-axis inertial measurement unit (IMU) and a temperature sensor. The device captures ECG at 128 Hz and IMU at 13 Hz. The device is operated on a lithium coin cell (CR-2025), that lasted approximately 400 h of recording. The Z3Pulse device transmits the data in real-time to a mobile app over Bluetooth low energy (BLE). Once recording is complete, the collected data is uploaded to the cloud and analyzed using Neurobit-HRV. A graphical summary of the different steps of the methodology, from data collection to automated sleep stage scoring, is displayed in Figure 2.

The process of aligning the data across PSG and Z3Pulse was essential to allow accurate comparison on an epoch-by-epoch basis. The timestamps of both systems were first synchronized using an internet time server as reference. Bedtimes for the PSG were estimated from the sleep diary. For Z3Pulse, position data were used to derive times on and off the bed. The time recorded from either the PSG or Z3Pulse, depending on the device with earlier lights-off time, was used as the reference data point. The shorter recording was extended to match the longer one by assuming “awake time” for missing epochs. An example of the scenario described is summarized in Supplementary Figure 3 in the Supplement.

### Data analysis

Firstly, we evaluated the classification performance of the 2-, 3-, and 4-levels models in both datasets considering each scored epoch as an independent observation. These epoch-level results are presented in the Supplementary material. The goodness of fit metrics computed were the following: Accuracy (A), Cohen’s kappa (K), Sensitivity (SE), Specificity (SP), Positive Predictive Value (PPV), and Negative Predicted Value (NPV). Then, the estimates obtained at the epoch level were averaged within each individual participant to derive subject-level results. Lastly, these subject-level results were averaged to cohort-level metrics. The described approach was conducted independently for the two cohorts analyzed.

The wealth of data within the CinC dataset allowed for performance evaluation of the algorithm as a function of factors known to have an impact on HR: age, AHI score, and biological sex. Participants’ ages and AHI scores were independently stratified using 3-level categorization; age ≤ 40 years-old, 40 < age ≤ 60 years-old, or age > 60 years-old and AHI ≤ 5 (None/Minimal) or 5 < AHI ≤ 15 (Mild) or AHI > 15 (Severe). Multiple independent linear regression models were used to estimate the association between age, AHI score, and biological sex on accuracy, kappa, SE, SP, PPV, and NPV. The limited sample size of the Z3Pulse dataset did not allow for analyses stratified by age, AHI score, and biological sex.

## Results

### Populations

For the CinC dataset a total of 6 participants were excluded from the analyses accordingly to the exclusion criteria illustrated in the previous sections. The final dataset included 988 subjects. There were 664 (∼67%) males, 324 (∼33%) females; 137 (∼14%), 285 (∼29%) and 566 (∼57%) participants in the None/Minimal, Mild, and Severe AHI groups, respectively; 157 (∼16%), 456 (∼46%) and 375 (∼38%) participants in the age ≤ 40 years-old, 40 < age ≤ 60 years-old, and age > 60 years-old groups, respectively. In the CinC cohort, participants’ total sleep time was 369.6 ± 69.2 (mean ± std) minutes, time spent in NREM sleep was 310.8 ± 59.8 min, and time spent in REM sleep was 58.8 ± 35.1 min.

For the Z3pulse dataset, a total of 44 (∼28%) out of 156 records were excluded. Most of the recordings of insufficient quality had missing data due to issues in data transmission rather than presenting excessive noisiness of the ECG traces. There were 27 (∼52%) males, 25 (∼48%) females; 38 (∼73%), 7 (∼13%) and 7 (∼13%) participants in the age ≤ 40 years-old, 40 < age ≤ 60 years-old, and age > 60 years-old groups, respectively. In the Z3pulse cohort, participants’ total sleep time was 402.5 ± 64.6 (mean ± std) minutes, time spent in NREM sleep was 311.1 ± 49.4 min, and time spent in REM sleep was 91.4 ± 30.6 min. A total of 112 recordings were included in the analysis.

Clinical and demographic data of the CinC and Z3pulse datasets are illustrated in Table 1.

**TABLE 1:** Summary characteristics of the participants included in CinC and Z3Pulse datasets.

	CinC	Z3Pulse
Participants included [count]	988	52
Recordings analyzed [count]	988	112
Biological Sex [male count (%age)]	664 (67.21%)	27 (51.92%)
Age ≤ 40 years-old [count (%age)]	137 (13.78%)	38 (73.08%)
40 < Age ≤ 60 years-old [count (%age)]	285 (28.84%)	7 (13.46%)
Age > 60 years-old [count (%age)]	566 (57.29%)	7 (13.46%)
AHI none/minimal [count (%age)]	157 (15.89%)	N/A
AHI mild [count (%age)]	456 (46.15%)	N/A
AHI severe [count (%age)]	375 (37.96%)	N/A
Total sleep time (mean ± std) [min]	369.6 ± 69.2	402.5 ± 64.6
Time spent in NREM sleep (mean ± std) [min]	310.8 ± 59.8	311.1 ± 49.4
Time spent in REM sleep (mean ± std) [min]	58.8 ± 35.1	91.4 ± 30.6

AHI score was not available (N/A) for participants included in the Z3Pulse dataset.

### Computing in Cardiology (CinC) dataset

**FIGURE 3:**
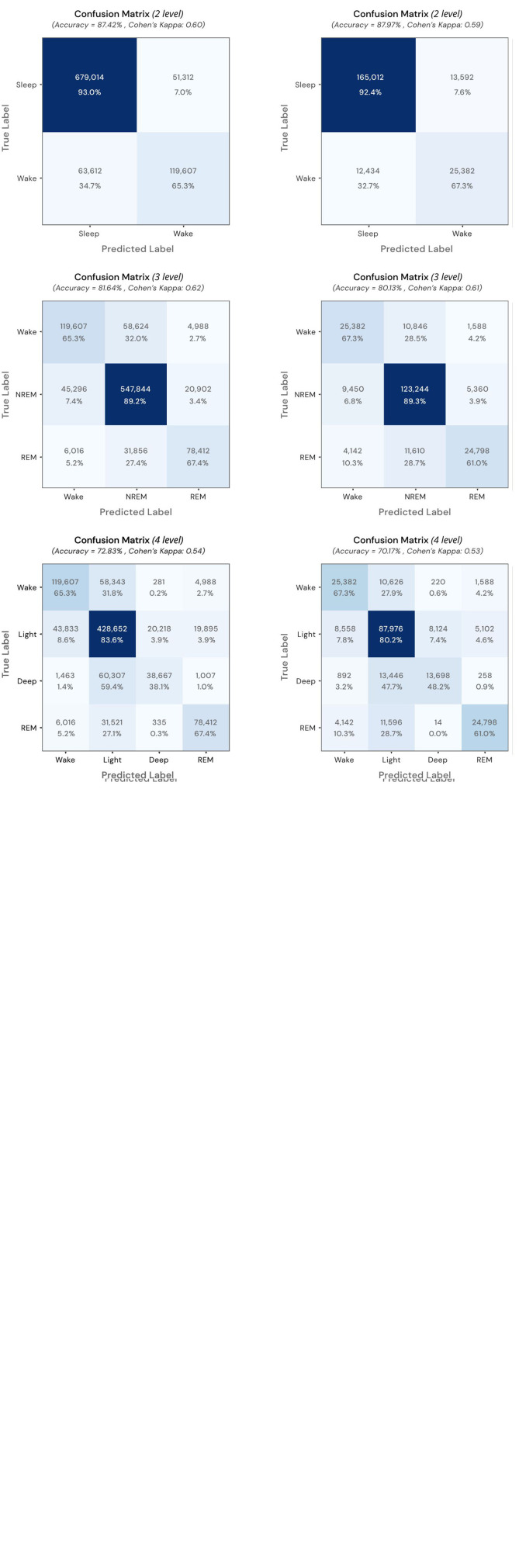
Full-counts and normalized confusion matrices for results of the 2-, 3-, and 4-levels prediction for the CinC dataset **(Left column)** and Z3Pulse dataset **(Right column)**.

Results for the 2-, 3-, and 4-levels prediction for the CinC dataset data are displayed in Figure 3 via confusion matrices and reported in Supplementary Table 1. The highest value of accuracy was achieved by the 2-levels classification (wake vs sleep), whereas the predictions obtained in the 3-levels model obtained the highest value of kappa. As described in the Methods section, by design of the algorithm, all the derived metrics (SE, SP, PPV, and NPV) relative to the classification of wake segments, are identical across the 2-, 3-, and 4-levels models. On the other hand, the classification of sleep segments is achieved by progressively collapsing different sleep states passing from the 4-levels to the 2-levels model. As a consequence, values of PPV and NPV were substantially equivalent across models and sleep states. The granular level of classification achieved by the 4-levels model obtained the lowest value of SE (0.3812) and the highest value of SP (0.9744) for the classification of DEEP sleep segments. The comparison of the goodness of fit metrics at the segment level (Supplementary Table 1) versus the ones obtained at the participant level (Table 2) revealed a close concordance between the two approaches.

**TABLE 2:** Mean and [95% Confidence Intervals] Accuracy (A), Cohen’s kappa (K), Sensitivity (SE), Specificity (SP), Positive Predictive Value (PPV), and Negative Predicted Value (NPV) were obtained by firstly collapsing metrics within and subsequently across participants in the CinC dataset.

CinC – Participants								
		2-levels			3-levels			4-levels
M O D E L	*A*	0.8797 [0.8740 0.8846]			0.8206 [0.8150 0.8262]			0.7295 [0.7232 0.7358]
	*K*	0.5754 [0.5634 0.5874]			0.6025 [0.5914 0.6136]			0.5122 [0.5020 0.5226]

	*SE*	0.6604 [0.6469 0.6739]	W A K E	*SE*	0.6688 [0.6555 0.6820]	W A K E	*SE*	0.6688 [0.6555 0.6820]
W A	*SP*	0.9309 [0.9257 0.9362]		*SP*	0.9116 [0.9086 0.9231]		*SP*	0.9116 [0.9086 0.9231]
K E	*PPV*	0.7093 [0.6960 0.7227]		*PPV*	0.7110 [0.6979 0.7143]		*PPV*	0.7110 [0.6979 0.7143]
	*NPV*	0.9171 [0.9116 0.9226]		*NPV*	0.9080 [0.9017 0.9143]		*NPV*	0.9080 [0.9017 0.9143]

S L E E P	*SE*	0.9309 [0.9257 0.9362]	R E M	*SE*	0.6494 [0.6304 0.6684]	R E M	*SE*	0.6494 [0.6304 0.6684]
	*SP*	0.6604 [0.6469 0.6739]		*SP*	0.9671 [0.9644 0.9697]		*SP*	0.9671 [0.9644 0.9697]
	*PPV*	0.9171 [0.9116 0.9226]		*PPV*	0.7493 [0.7335 0.7651]		*PPV*	0.7493 [0.7335 0.7651]
	*NPV*	0.7093 [0.6960 0.7227]		*NPV*	0.9528 [0.9498 0.9558]		*NPV*	0.9528 [0.9498 0.9558]
	
S L E E P			N R E M	*SE*	0.8949 [0.8890 0.9008]	L I G H T	*SE*	0.8163 [0.8073 0.8253]
				*SP*	0.7042 [0.6927 0.7157]		*SP*	0.6357 [0.6251 0.6464]
				*PPV*	0.8637 [0.8576 0.8698]		*PPV*	0.7478 [0.7395 0.7561
				*NPV*	0.7705 [0.7608 0.7802]		*NPV*	0.7463 [0.7368 0.7559]
			
S L E E P						D E E P	*SE*	0.3831 [0.3628 0.4034]
							*SP*	0.9612 [0.9563 0.9661
							*PPV*	0.6444 [0.6211 0.6677]
							*NPV*	0.9238 [0.9196 0.9281]

Results are reported separately for the 2-, 3- and 4-levels models.

Table 3 summarizes the performance of the 2-levels model as a function of participants’ characteristics. AHI and biological sex groups did not affect the outcome metrics. In contrast, a significant decrease in A and K were reported for older age groups when compared to the reference age group (age ≤ 40 years-old). Specifically, the average decrease in A for the age group 40 < Age ≤ 60 and Age > 60 was 3.02 and 7.45%, respectively. Similar results were found for the other outcome metrics such that the mean decrease in performance in the group of participants age > 60 years old is approximately double that of the 40 < age ≤ 60 group. Analogous findings were reported when considering the 3-levels and 4-levels models as reported in Supplementary Tables 2, 3.

**TABLE 3:** Mean and [95% confidence intervals] of beta estimates of the performance of the 2-levels models as a function of the participants’ characteristics: age, AHI score, and biological sex.

		40 < Age ≤ 60	Age > 60	5 < AHI ≤ 15	AHI > 15	Sex (M)
M O D E L	*A*	–0.0302*** [–0.0449 -0.0156]	–0.0745*** [–0.0897-0.0593]	–0.0009^n.s.^	–0.0103^n.s.^	–0.0025^n.s.^
	*K*	–0.0657*** [–0.0998 -0.0315]	–0.1091*** [–0.1446 -0.0736]	–0.0124^n.s.^	–0.0335^n.s.^	0.0055^n.s.^

W A K E	*SE*	–0.0453* [–0.0841 -0.0066]	–0.1042*** [–0.1443 -0.0638]	–0.0016^n.s.^	–0.0120^n.s.^	0.0126^n.s.^
	*SP*	–0.0158* [–0.0312 -0.0005]	–0.0267** [–0.0426 -0.0108]	–0.0057^n.s.^	–0.0140^n.s.^	–0.0051^n.s.^
	*PPV*	–0.0394* [–0.0782 -0.0005]	0.0099^n.s.^	–0.0017^n.s.^	–0.0407^n.s.^	–0.0089^n.s.^
	*NPV*	–0.0220** [–0.0370 -0.0070]	–0.0767*** [–0.0924 -0.0611]	0.0079^n.s.^	0.0026^n.s.^	0.0045^n.s.^

The reported estimates for beta were obtained by independently testing the association between age, AHI score, biological sex, and each figure of merit. In the conducted linear regression models, the reference groups were Age ≤ 40, AHI ≤ 5, and female biological sex. n.s. = not significant, p-value ≥ 0.05; *p-value < 0.05; **p-value < 0.01; ***p-value < 0.001.

### Z3Pulse dataset

Results for the 2-, 3-, and 4-levels prediction are reported in Table 4 (at the participant level) and Supplementary Table 4 (at the segment level). The highest value of accuracy was achieved by the binary classification (wake vs sleep) performed in the 2-levels model (0.8812 [0.8668–0.8955] and 0.8797, at the participant and segment levels, respectively), whereas the prediction obtained in the 3-levels model obtained the highest value of kappa [0.611 (0.5818, 0.6401) and 0.6117]. Regarding the classification of the sleep states, the lowest value of SE [0.6163 (0.5710, 0.6617) and 0.6115] and the highest value of SP [0.9606 (0.9536, 0.9676) and 0.9605] were obtained across the classification of DEEP sleep segment. Values of PPV and NPV were comparable equivalent across models and sleep states. The comparison of the goodness of fit metrics at the segment (Table 4) versus that obtained at the participant level (Supplementary Table 4) revealed a close concordance across the different metrics.

**TABLE 4:** Mean and [95% confidence Intervals] Accuracy (A), Cohen’s kappa (K), Sensitivity (SE), Specificity (SP), Positive Predictive Value (PPV), and Negative Predicted Value (NPV) were obtained by firstly collapsing metrics within and subsequently across participants in the Z3Pulse dataset.

Z3Pulse – Participants								
		
		2-levels			3-levels			4-levels
M O D E L	*A*	0.8812 [0.8668 0.8955]			0.8035 [0.7871 0.8199]			0.7030 [0.6869 0.7192]
	*K*	0.5882 [0.5558 0.6206]			0.6110 [0.5818 0.6401]			0.5254 [0.5000 0.5508]
	
	*SE*	0.7125 [0.6796 0.7454]	W A K E	*SE*	0.7125 [0.6796 0.7454]	W A K E	*SE*	0.7125 [0.6796 0.7454]
W A	*SP*	0.9240 [0.9113 0.9368]		*SP*	0.9240 [0.9113 0.9368]		*SP*	0.9240 [0.9113 0.9368]
K E	*PPV*	0.6672 [0.6287 0.7057]		*PPV*	0.6672 [0.6287 0.7057]		*PPV*	0.6672 [0.6287 0.7057]
	*NPV*	0.9314 [0.9160 0.9468]		*NPV*	0.9314 [0.9160 0.9468]		*NPV*	0.9314 [0.9160 0.9468]

S L E E P	*SE*	0.9240 [0.9113 0.9368]	R E M	*SE*	0.6163 [0.5710 0.6617]	R E M	*SE*	0.6163 [0.5710 0.6617]
	*SP*	0.7125 [0.6796 0.7454]		*SP*	0.9606 [0.9536 0.9676]		*SP*	0.9606 [0.9536 0.9676]
	*PPV*	0.9314 [0.9160 0.9468]		*PPV*	0.7765 [0.7419 0.8111]		*PPV*	0.7765 [0.7419 0.8111]
	*NPV*	0.6671 [0.6287 0.7057]		*NPV*	0.9177 [0.9075 0.9278]		*NPV*	0.9177 [0.9075 0.9278]
	
S L E E P			N R E M	*SE*	0.8917 [0.8771 0.9063]	L I G H T	*SE*	0.8009 [0.7824 0.8194]
				*SP*	0.7264 [0.7007 0.7521]		*SP*	0.6728 [0.6490 0.6965]
				*PPV*	0.8505 [0.8332 0.8678]		*PPV*	0.7203 [0.7002 0.7403]
				*NPV*	0.7956 [0.7742 0.8170]		*NPV*	0.7665 [0.7474 0.7855]
			
S L E E P						D E E P	*SE*	0.4755 [0.4214 0.5297]
							*SP*	0.9544 [0.9451 0.9638]
							*PPV*	0.6102 [0.5544 0.6659]
							*NPV*	0.9254 [0.9143 0.9365]

Results are reported separately for the 2-, 3- and 4-levels models.

## Discussion

The aim of this study was to validate the performance of the previously trained and tested Neurobit-HRV deep learning model for automatic sleep staging, utilizing two datasets, namely a publicly available collection of PSG recordings [You Snooze You Win – The PhysioNet CinC Challenge 2018 dataset (Goldberger et al., 2000)], and a proprietary dataset of simultaneously collected PSG and single lead ECG acquired using a wearable device (Z3Pulse device). The results of the classification performance in the two datasets highlighted high and consistent accuracy, 88, 82, and 73% for the 2- levels, 3- levels and 4-level models, respectively, in the CinC dataset and 88, 80, and 70% for the Z3Pulse dataset. In addition, the agreement of such metrics compared to results obtained in the training/testing phase (see Figure 1), speaks to the robustness of the developed framework. In fact, the accuracy of the 4-level model in the original training (75.15%) and testing set (70.71%) is comparable to the results obtained in the CinC (72.95%) and Z3Pulse (70.30%) datasets.

Recent years have seen the surge of automated sleep stage algorithms leveraging a variety of physiological signals collected via traditional, semiportable, or wearable devices. While models trained on the entirety of the physiological signals collected via PSG are able to achieve performance close to human scorers (Vallat and Walker, 2021), the interest in quantifying and tracking human sleep by means of less invasive and easy deployable devices has increased exponentially. Therefore, the demand for automated, accurate, and highspeed algorithms working with a reduced set of features has scaled accordingly. Proposed models have evolved from simple binary detection of sleep–wake to a more fine-grained sleep classification. Examples are automatic frameworks utilizing a single lead EEG, either isolated from PSG recordings or collected using the Zmachine^®^ sleep monitoring system (Wang et al., 2015; Yoon et al., 2017b; Olesen et al., 2021), or a single lead ECG either isolated from PSG studies or acquired using a variety of strategies (Li et al., 2018; Walch et al., 2019; Sridhar et al., 2020) potentially complemented by respiratory effort (Fonseca et al., 2015; Bakker et al., 2021). The model validated in this study provides a significant contribution toward the methodological advancement of automatic sleep stage scoring relying solely on a single lead ECG signal, acquired via a wearable patch. In addition, the algorithm solely utilizes HR hence it is independent of the ECG morphology. As such, the proposed solution algorithm can easily work with other sensor modalities which are capable of measuring HR, irrespective of the original acquired signal. This is an important consideration, given the rapid proliferation of internet of things (IoT) devices capable of measuring HR through various contact and non-contact sensors. Furthermore, to fully describe the performance of the algorithm, we calculated several goodness of fit in addition to accuracy, such as kappa, SP, SE, NPV and PPV not only for the overall models but for each of the predicted sleep states. Although these values are inextricably linked, they do provide complementary information (Trevethan, 2017) and allow for a better assessment of the performance of the algorithm.

In this work, we show that the proposed model can not only achieve high accuracy, but also obtained adequate performance on other metrics across multiple datasets not employed for training nor testing. The achieved results are comparable with previous work by other research groups. Sridhar et al. developed an automated sleep staging algorithm using instantaneous heart rate (Sridhar et al., 2020). Analogously to our approach, the CinC dataset was utilized to validate their previously trained and tested architecture. Values of accuracy and kappa are substantially equivalent to our results; accuracy was equal to 0.72 in both methods, and we obtained a 0.01 lower value of kappa (kappa was equal to 0.54 in the reported analysis). Furthermore, both algorithms displayed the worst performance in the classification of light and deep sleep epochs. One possible explanation for the reported low value of sensitivity in deep sleep could be the inter-rater reliability of expert raters, since agreement in scoring deep sleep has been reported be the lowest and to vary significantly with age and biological sex (Danker-Hopfe et al., 2004; Rosenberg and Van Hout, 2013). Another relevant architecture was proposed by Radha et al. (2019). This solution utilizes parameters extracted from the heart rate variability signal. Whilst the obtained results of accuracy and kappa are slightly higher than those reported in this manuscript, it should be noted that the algorithm was validated on a considerably smaller sample size and not using an independent dataset. In addition, authors report a decrease in performance by age which is significantly more pronounced that the results reported in Table 3. Lastly, distributions of accuracy and kappa across sleep states reported by Radha et al. are characterized by a significantly higher variability compared to our findings. Interestingly, the model proposed in this manuscript achieved equivalent performance or even outperformed models utilizing ECG in combination with other physiological signals. As an example, the solution proposed by Sun et al. (2020) achieved similar performance despite pairing ECG with abdominal respirator effort. A similar approach was proposed by Rahimi et al. (2019) which combined heart rate variability parameters and ECG-derived respiration. Accuracy for the 2-level and 3-level models are approximately 10% lower compared to the results obtained in our validation study which solely utilized the ECG signal to derive sleep state classification. A summary of the comparisons between the proposed architecture and previous work published in the literature is reported in Table 5.

**TABLE 5:** Summary of articles cited in the discussion section for the purpose of comparing the proposed solution with the existing literature.

Research study	Signal(s)	Signals/features	Sleep stages classified	Accuracy	Kappa	Validation	Participant	Age	Location	Data source
Abou Jaoude et al., 2020	EEG	4 channels	Wake-N1-N2-N3-REM	77.3%	0.69	PSG	243	>18	Hospital	HomePAP Database
Bakker et al., 2021	ECG + RIP	HR + Respiration	Wake-N1-N2-N3-REM	78.00%	0.64	PSG	296	50-90	Hospital	National Sleep Research Resource
Fonseca et al., 2016	ECG + RIP	RRI + Respiration	Wake-REM-Light-Deep	87.40%	0.41	PSG	180	20–95	Hospital	Multiple Databases
Fonseca et al., 2017	ACC + PPG	HRV + Movement	Wake-N1-(N2+N3)-REM	59.3%	0.42	PSG	51	41–60	Home	Alice PDx
Fonseca et al., 2020	ECG + ACC	HRV + Movement	Wake-N1-(N2+N3)-REM	75.9%	0.60	PSG	389	–	Hospital	SOMNIA Dataset
Li et al., 2018	ECG	HRV + EDR	Wake-REM-Light-Deep	75.4%	0.54	PSG	16	32–56	Hospital	MIT-BIH
Li et al., 2020	ACC	Movement	Wake-Sleep	84.7%	0.45	PSG	43	45–84	Sleep Lab	MESA Sleep Dataset
Long et al., 2013	RIP + ACC	Effort + Movement	Wake-Sleep	95.7%	0.66	PSG	15	23–58	Sleep Lab	Actiwatch
Quante et al., 2018	ACC	Movement	Wake-Sleep	85.0%	–	PSG	22	20–45	Home	GT3X
Shen et al., 2019	EEG	1 channel	Wake-N1-N2-N3-REM	81–92%	0.89	PSG	48	20–65	Hospital	Sleep EDF + DREAMS
Sridhar et al., 2020	ECG	HR	Wake-REM-Light-Deep	77%	0.66	PSG	–	–	Sleep Lab	MESA sleep + SHHS
Walch et al., 2019	PPG + ACC	HR + Movement	Wake-NREM-REM	72%	0.27	PSG	31	19–55	Hospital	Apple watch
Wang et al., 2012	ECG	HRV	Wake-NREM-REM	73.50%	–	PSG	7 (OSA)	42–68	Hospital	Unknown
Wang et al., 2015	EEG	1 channel	Wake-REM-Light-Deep	–	0.72	PSG	99	18–60	Sleep Lab	Zmachine
Wei et al., 2018	EEG	1 channel	Wake-NREM-REM	77%	0.56	PSG	16	32–56	Hospital	MIT-BIH
Wei et al., 2019	ECG	HRV + RRV	Wake-N1-N2-N3-REM	71.16%	0.52	PSG	373	22–56	Hospital	SOLAR 3000B
Yoon et al., 2017a	ECG	RRI	N3	90%	0.56	PSG	45 (OSA)	–	Hospital	NI DAQ 6221
Zhang et al., 2012	PPG + ACC	HRV + Movement	Wake-NREM-REM	75%	–	PSG	48	22–71	Hospital	Research Device

ACC, accelerometer; ECG, electrocardiography; EDR, ECG-derived respiration; EEG, electroencephalography; HR, heart rate; HRV, heart rate variability; PPG, photoplethysmography; RIP, respiratory inductive plethysmography; RRI, R-R Intervals.

Concurrently, recent years have seen the exponential growth of PPG-based wearables aimed to perform sleep stage classification in the natural/home environment. As recently summarized in a comprehensive systematic review (Imtiaz, 2021), PPG-based solutions are on average easier to use and better suited for wearable/nearable monitoring but often unable to reliably characterize the full spectrum of sleep stages. A recent manuscript by Zhao and Sun (2021), proposed an approach similar to our work despite limited to a significantly smaller sample size. The reported results for accuracy and Cohen’s kappa of the 3-level and 4-level models are aligned with our findings. Compared to most of the available PPG-based solutions for sleep classification, the model presented by Zhao et al. only uses a single-channel PPG signals as classification data and does not require the use of complementary information from other signals. However, the authors do not report other metrics of goodness of fit nor performance of the 2-level classification. An additional insightful approach into the PPG-based approaches is presented by Li et al. (2021). The authors developed a sleep staging method using wrist-worn accelerometry in combination with PPG, leveraging transfer learning from a large database of ECG recordings (5,793 participants). Transfer learning was applied to train a PPG-based model by taking advantage of the extant approaches built from massively available data type such as ECG. When considering PPG data only, the values of accuracy for the 2-, 3- and 4-level models are approximately 0.10 lower compared to the presented model, whereas the values of Cohen’s kappa are 1.5 times lower. Similar results are obtained when models were trained by combining PPG and actigraphy data. A comprehensive review of the most recent developed ECG- and PPG-based models is presented in (Imtiaz, 2021).

One additional advantage of the proposed algorithm is that it does not require contextual information, such as start time of the recording, thus being agnostic and removing any potential sources of bias. This type of bias is known to affect algorithms to analyze actigraphy data (Paquet et al., 2007) and manual PSG scoring. We also tested if other demographic factors known to be associated with sleep characteristics, namely biological sex, age, and AHI, would affect algorithm performances. We showed the insensitivity of the algorithm to both AHI and biological sex. Such characteristics have been previously reported to affect classification performance (Redline et al., 2004) thus, our methodology is seen as an advancement toward a universally applicable sleep scoring model. However, our results showed that the performance of the algorithm was associated with participants’ age. One explanation for this finding may be attributed to the differences in age group distributions between the training/testing versus the validation sets. Extensive literature has shown that HR indices parameters change with age (Voss et al., 2015), thus for future development we anticipate additional data collection across various age groups to optimize the algorithm.

Lastly, the algorithm and datasets utilized in this manuscript are publicly available. This is an essential step to support wide adoption of automatic sleep staging algorithms within the research and clinical communities.

## Limitations

The main limitation of the proposed algorithm is the lack of interpretability of the information extracted from the input HR signal due to the black box nature of deep-learning methods (Samek et al., 2017; Shah and Chircu, 2018; Bartoletti, 2019; Panch et al., 2019; Panesar, 2019; Adadi and Berrada, 2020; Stiglic et al., 2020). Given that HR segments are directly fed into the model, it is non-trivial to gain insight onto the features extracted at the level of encoding/decoding layers nor identify those contributing the most to predicting the sleep stage membership of each scored segment. While advances in machine learning have enables visualization, interpretability, and explicability of the trained architectures (Samek et al., 2017; Panch et al., 2019; Panesar, 2019; Stiglic et al., 2020), an in depth characterization of the features extracted by the network is beyond the scope of the present study. In future work, it would be desirable to compare such features with time, frequency, and nonlinear features widely employed in the HRV literature. Recent years have seen the development of a variety of artificial intelligence architecture characterized by interpretable knowledge representations (without any additional human supervision) (Seo et al., 2017; Zhang et al., 2018; Adadi and Berrada, 2020). Experiments have shown that our interpretable artificial intelligence and machine learning methodologies encoded more semantically meaningful knowledge in high dimensional convolutional layers than traditional architectures (Zhang et al., 2018; Kuo et al., 2019). Dimensionality reduction techniques have been proposed to gain insight into the complex sequence of spatial-spectral filtering operations performed by the convolutional layers. A widely exploited transformation is called Saab (Subspace approximation with adjusted bias) (Kuo et al., 2019). The Saab method is a variant of PCA, and it contributes to dimension reduction. This operation enhances discriminability of some dimensions by deriving dimensions with a larger receptive which are intrinsically less granular and easier to characterize.

Further technological improvement of Z3Pulse system is warranted. As the scenarios of utilization rapidly expand, the system may undergo additional modification of the hardware, firmware, and software. In fact, the current version of the system has benefited immensely from the testing conducted outside of traditional clinical settings. The initial version of the Z3pulse relied exclusively on a continuous BLE connection between the device and the phone. This characteristic presented a significant limitation, as multitude of instances may terminate such connectivity. Examples are the mobile operating system terminating the app without notice due to power or resource constraint, the connection interrupted in instances when the participants were outside of phone’s Bluetooth range. This resulted in a considerable portion of the recordings failing quality checks. Informed by the data collected in low-middle income settings as well as the consideration above-listed, the RR detection and SNR calculation algorithms were moved into the device firmware. As such, the device can record the HR, SNR, and position directly on the device itself. In the current configuration, the phone is used to signal the device for starting the recording and retrieve the data once data acquisition is completed.

## Conclusion

In conclusion, the solution illustrated in this manuscript highlight the opportunity of utilizing an inexpensive and widely available ECG wearable devices paired with automated sleep staging algorithms to characterize sleep architecture rapidly and inexpensively with high levels of accuracy. Moreover, the reported findings set the foundation for a HR-operated (derived from gold-standard ECG) algorithm. By benefiting from the open and accessible nature of the algorithm, the proposed solution can be virtually integrated with any sensors capable of estimating HR. In the future, we anticipate utilizing the described algorithm on HR data derived from other sensors as well as comparing the robustness of the proposed solution. This work offers the opportunity to enable innovative scalable and accessible modalities for monitoring sleep in the home environment, increasing equitable access to sleep health.

## Data availability statement

The CinC dataset analyzed for this study can be found in the You Snooze You Win-The PhysioNet Computing in Cardiology (CinC) Challenge 2018 dataset. The dataset is available at: https://physionet.org/content/challenge-2018/1.0.0/. The Z3Pulse dataset generated and analyzed for this study is not publicly available. The access to the Z3Pulse dataset is managed by AP. Data may be shared upon reasonable request addressed to NP, AP, and ML.

## Ethics statement

The studies involving human participants were reviewed and approved by National University of Singapore’s Institutional Review Board (NUS-IRB). The patients/participants provided their written informed consent to participate in this study.

## Author contributions

NP, AP, WF, and ML were involved in study design, literature search, validation, data curation, data analysis, and wrote the manuscript. NP, JO, NC, ZS, AA, SB, and KK organized the database, literature review, and prepared pictures and tables. All authors contributed to the interpretation of the results, manuscript revision and reviewed the final version making the necessary changes, approved the submitted version, and agreed to be accountable for the content of the work.

## Footnotes

1
http://research.neurobit.com/

2
https://gist.github.com/neurobittechnologies/e1d13e0b59c56f5804af7c127b36d20d

3
https://keras.io/

4
https://www.tensorflow.org/

5
www.movesense.com
